# Full genome characterization of novel DS-1-like G9P[8] rotavirus strains that have emerged in Thailand

**DOI:** 10.1371/journal.pone.0231099

**Published:** 2020-04-22

**Authors:** Saori Fukuda, Ratana Tacharoenmuang, Ratigorn Guntapong, Sompong Upachai, Phakapun Singchai, Tomihiko Ide, Riona Hatazawa, Karun Sutthiwarakom, Santip Kongjorn, Napa Onvimala, Kriangsak Ruchusatsawast, Pimpa Rungnopakun, Jutarat Mekmallika, Yoshiki Kawamura, Kazushi Motomura, Masashi Tatsumi, Naokazu Takeda, Takayuki Murata, Tetsushi Yoshikawa, Ballang Uppapong, Koki Taniguchi, Satoshi Komoto

**Affiliations:** 1 Department of Virology and Parasitology, Fujita Health University School of Medicine, Toyoake, Aichi, Japan; 2 National Institute of Health, Department of Medical Sciences, Nonthaburi, Thailand; 3 Department of Pediatrics, Fujita Health University School of Medicine, Toyoake, Aichi, Japan; 4 Center for Research Promotion and Support, Joint Research Support Promotion Facility, Fujita Health University, Toyoake, Aichi, Japan; 5 Bhumibol Adulyadej Hospital, Bangkok, Thailand; 6 Thailand-Japan Research Collaboration Center on Emerging and Re-emerging Infections, Nonthaburi, Thailand; 7 Osaka Institute of Public Health, Osaka, Japan; Universita degli Studi di Parma, ITALY

## Abstract

The emergence and rapid spread of unusual DS-1-like intergenogroup reassortant rotaviruses having G1/3/8 genotypes have been recently reported from major parts of the world (Africa, Asia, Australia, Europe, and the Americas). During rotavirus surveillance in Thailand, three novel intergenogroup reassortant strains possessing the G9P[8] genotype (DBM2017-016, DBM2017-203, and DBM2018-291) were identified in three stool specimens from diarrheic children. In the present study, we determined and analyzed the full genomes of these three strains. On full-genomic analysis, all three strains were found to share a unique genotype constellation comprising both genogroup 1 and 2 genes: G9-P[8]-I2-R2-C2-M2-A2-N2-T2-E2-H2. Phylogenetic analysis demonstrated that each of the 11 genes of the three strains was closely related to that of emerging DS-1-like intergenogroup reassortant, human, and/or locally circulating human strains. Thus, the three strains were suggested to be multiple reassortants that had acquired the G9-VP7 genes from co-circulating Wa-like G9P[8] rotaviruses in the genetic background of DS-1-like intergenogroup reassortant (likely equine-like G3P[8]) strains. To our knowledge, this is the first description of emerging DS-1-like intergenogroup reassortant strains having the G9P[8] genotype. Our observations will add to the growing insights into the dynamic evolution of emerging DS-1-like intergenogroup reassortant rotaviruses through reassortment.

## Introduction

Group A rotavirus (RVA) within the *Reoviridae* family, is the primary pathogen that causes severe gastroenteritis in young children and animals worldwide. RVA disease is responsible for an estimated 128,500–215,000 deaths among children <5 years of age annually [1, 2]. The RVA genome consists of 11 segments of double-stranded (ds)RNA, encoding six structural proteins (VP1-VP4, VP6, and VP7) and six non-structural proteins (NSP1-NSP6) [3]. The segmented nature of the genome facilitates reassortment between/among RVA strains, and the reassortment plays one of the major roles in the dynamic evolution of RVAs [4].

RVAs have been traditionally differentiated by means of a binary classification system based on their two outer capsid proteins, VP7 and VP4, which are independently involved in viral neutralization, and define the G and P genotypes, respectively. Thus far, RVAs have been classified into at least 36 G and 51 P genotypes (https://rega.kuleuven.be/cev/viralmetagenomics/virus-classification). Among them, 6 G (G1-G4, G9, and G12) and 3 P (P[4], P[6], and P[8]) genotypes are considered as common genotypes of human RVAs (HuRVAs), although there are some differences in geographic distribution [3]. Since 2008, a full genome-based genotyping system based on assignment of all the 11 gene segments has been introduced, where Gx-P[x]-Ix-Rx-Cx-Mx-Ax-Nx-Tx-Ex-Hx designates the genotypes of the VP7-VP4-VP6-VP1-VP2-VP3-NSP1-NSP2-NSP3-NSP4-NSP5 genes [5, 6]. Most HuRVA strains are divided into a Wa-like (genogroup 1) or DS-1-like (genogroup 2) genotype constellation [6, 7]. The Wa-like strains have the backbone genomic constellation I1-R1-C1-M1-A1-N1-T1-E1-H1 and tend to have G/P genotypes, G1P[8], G3P[8], G4P[8], G9[8], and G12P[8], whereas the DS-1-like strains have backbone genomic constellation I2-R2-C2-M2-A2-N2-T2-E2-H2 and tend to have G2P[4] [6, 8]. Although intergenogroup reassortment can occur, it is generally believed that intergenogroup reassortant strains have an evolutionary fitness disadvantage compared to the parental Wa-like and DS-1-like strains, and thus would be selected against in nature [6, 7, 9]. Nevertheless, the emergence and rapid spread of uncommon DS-1-like intergenogroup reassortant strains, i.e., DS-1-like G1P[8] strains and their derivatives having genotype constellations G1/3/8-P[8]-I2-R2-C2-M2-A2-N2-T2-E2-H2, have been recently reported from major parts of the world (Africa, Asia, Australia, Europe, and the Americas) [6, 10–34].

The first DS-1-like intergenogroup reassortant strains, i.e., DS-1-like G1P[8] strains, were identified in diarrheic children in Japan in 2012 [15, 24, 33], and subsequently DS-1-like G1P[8] strains were reported from Thailand, Brazil, Malawi, the Philippines, and Vietnam in 2012–2013 [18, 21, 25, 27, 33], and Pakistan in 2016 [30]. In 2013, DS-1-like (equine-like) G3P[8] strains emerged in Thailand and Australia [12, 22], and equine-like G3P[8] strains have successfully spread to several parts of the world, i.e., Dominica in 2014 [18], Germany, Hungary, Indonesia, Japan, Spain, and the United States in 2015 [6, 10, 13, 20, 28, 29, 32], Brazil in 2016 [16], and Italy in 2018 [14]. In 2013, DS-1-like (bovine-like) G8P[8] strains also emerged in Thailand [31], and subsequently bovine-like G8P[8] strains were detected in the Czech Republic, Japan, and Vietnam in 2014 [17, 23, 26], and Singapore in 2016 [11].

In 2017–2018, we detected five novel DS-1-like intergenogroup reassortant strains having the G9P[8] genotype with a short electropherotype in diarrheic children in Thailand, a total of 429 RVA-positive stool specimens being examined by RT-PCR-based G/P genotyping and polyacrylamide gel electrophoresis (PAGE) analysis during the RVA surveillance in 2015–2018 (Tacharoenmuang et al., in preparation), while no DS-1-like G9P[8] strain was detected in 2015–2016. Because these DS-1-like G9P[8] strains were unusual, full-genomic analysis of these Thai strains might be useful for obtaining a more precise understanding of the evolutionary dynamics of emerging DS-1-like intergenogroup reassortant strains. In the present study, we sequenced and characterized the full genomes of three representative DS-1-like G9P[8] strains that have emerged in Thailand.

## Materials and methods

### Ethics statement

This study was approved by the Ethical Review Committee for Research on Human Subjects, Ministry of Public Health, Thailand (Ref. no. 0032/2556). In this study, written informed consent for the testing of stool specimens for RVAs and characterization of detected RVA strains was obtained from the children’s parents/guardians. Questionnaire information was deidentified and re-coded so that no information could be linked to any individual participant.

### Virus strains

During the RVA surveillance program in Thailand in 2015–2018, which involved a total of 429 RVA-positive fecal specimens (Tacharoenmuang et al., in preparation), five G9P[8] strains with a short electropherotype were detected in stool samples from diarrheic children (aged 5 months to 5 years 5 months) admitted to Bhumibol Adulyadej Hospital in Bangkok. Out of the five identified G9P[8] strains with a short electropherotype, three representative strains showing intense genomic dsRNA bands on PAGE analysis were selected (strains DBM2017-016 and DBM2017-203 in 2017, and strain DBM2018-291 in 2018) for full genome-based analysis. In addition, the nucleotide sequences of the full genomes of five locally circulating HuRVA strains (three G2P[4] strains with a short electropherotype (DBM2017-003, DBM2017-015, and DBM2018-105) and two G9P[8] strains with a long electropherotype (DBM2017-014 and DBM2018-111)) detected in stool specimens from diarrheic children (aged 1 year 7 months to 10 years 5 months) admitted to Bhumibol Adulyadej Hospital were determined as well, as references. Stool samples containing the above-mentioned eight HuRVA strains were kept at −30°C until use.

### Viral genomic dsRNA extraction, cDNA library building, and Illumina MiSeq sequencing

RVA genomic dsRNAs were extracted from stool specimens using a QIAamp Viral RNA Mini Kit (Qiagen), and the dsRNAs were subjected to Illumina MiSeq sequencing as described previously [35, 36]. In brief, a 200 bp fragment library ligated with bar-coded adapters was built for the eight HuRVA strains using an NEBNext Ultra RNA Library Prep Kit for Illumina v1.2 (New England Biolabs), and NEBNext Multiplex Oligos for Illumina (New England Biolabs) according to the manufacturer’s instructions. The cDNA library was purified using Agencourt AMPure XP magnetic beads (Beckman Coulter). After assessing the quality and quantity of the purified cDNA library, nucleotide sequencing was performed on an Illumina MiSeq sequencer (Illumina) using a MiSeq Reagent Kit v2 (Illumina) to generate 151 paired-end reads. Bioinformatics analysis was carried out according to the protocol previously described [37]. Sequence reads were trimmed to exclude the adapters, primers, and low-quality sequences, using CLC Genomics Workbench v8.0.1 (CLC Bio). The parameter settings for the quality trimming were as follows: trim using quality scores, limit = 0.08; trim ambiguous nucleotides, maximum number of ambiguities = 4; and filter on length, discard reads below length = 15. Data analysis was performed using CLC Genomics Workbench v8.0.1. Contigs were assembled from the obtained sequence reads (trimmed) by *de novo* assembly. Using the assembled contigs as query sequences, the Basic Local Alignment Search Tool (BLAST) non-redundant nucleotide database was searched to determine which contig represents the full-length nucleotide sequence of each gene segment of the eight HuRVA strains. To further refine the contigs, the sequence reads of each gene were mapped back to the assembled contigs. The nucleotide sequences were translated into amino acid sequences using GENETYX v11 (GENETYX, Tokyo, Japan).

### Determination of RVA genotypes

The genotype of each of the 11 genes of the three DS-1-like G9P[8] and five G2P[4]/G9P[8] reference strains was determined using the RotaC v2.0 automated genotyping tool (http://rotac.regatools.be/) [38] according to the guidelines proposed by the Rotavirus Classification Working Group (RCWG) [39].

### Phylogenetic analysis

Multiple alignment of each gene was carried out using ClustalW. Maximum-likelihood phylogenetic trees were constructed for the 11 genes. The best substitution models for the 11 genes were decided based on the corrected Akaike information criterion value as implemented in MEGA7.0.26. The models used in this study were Tamura 3-parameter (T92) + gamma distributed (G) (VP7, VP4, and VP6), Tamura-Nei (TN93) + G + invariable sites (I) (VP1), TN93 + I (VP2), T92 + G + I (VP3), and T92 + I (NSP1-NSP5). The reliability of the branching was estimated from 1000 bootstrap replicates. For designating lineages, the already established lineages were referenced [40].

### Nucleotide sequence accession numbers

The nucleotide sequence data presented in this manuscript have been deposited in the DDBJ and EMBL/GenBank data libraries. The accession numbers for the nucleotide sequences of the VP1-VP4, VP6, VP7, and NSP1-NSP5 genes of strains DBM2017-016, DBM2017-203, DBM2018-291, DBM2017-003, DBM2017-015, DBM2018-105, DBM2017-014, and DBM2018-111 are LC514470-LC514480, LC514481-LC514491, LC514492-LC514502, LC514503-LC514513, LC514514-LC514524, LC514525-LC514535, LC514536-LC514547, and LC514548-LC514557, respectively.

## Results

### Nucleotide sequencing and whole genotype constellation

The genomic dsRNAs of three Thai G9P[8] strains with a short electropherotype, DBM2017-016, DBM2017-203, and DBM2018-291, were extracted from stool samples. Strains DBM2017-016, DBM2017-203, and DBM2018-291 were isolated from a 3 years 9 months old female, a 2 years old male, and a 5 months old female, respectively. None of the three children had received an RVA vaccination. To gain an insight into the genomic variety among the three strains, and the genetic relatedness with other RVA strains worldwide, full-genome sequencing of these three strains was performed using an Illumina MiSeq platform. In addition, the nucleotide sequences of the full genomes of five locally circulating HuRVA strains (three G2P[4] strains with a short electropherotype and two G9P[8] strains with a long electropherotype) were determined as well, as references. Complete or nearly complete nucleotide sequences of all 11 genes of these eight HuRVA strains could be determined. The lengths of the nucleotide and amino acid sequences of the 11 genes of the eight Thai RVA strains, with related sequence read data, are summarized in S1 Table.

**Fig 1:**
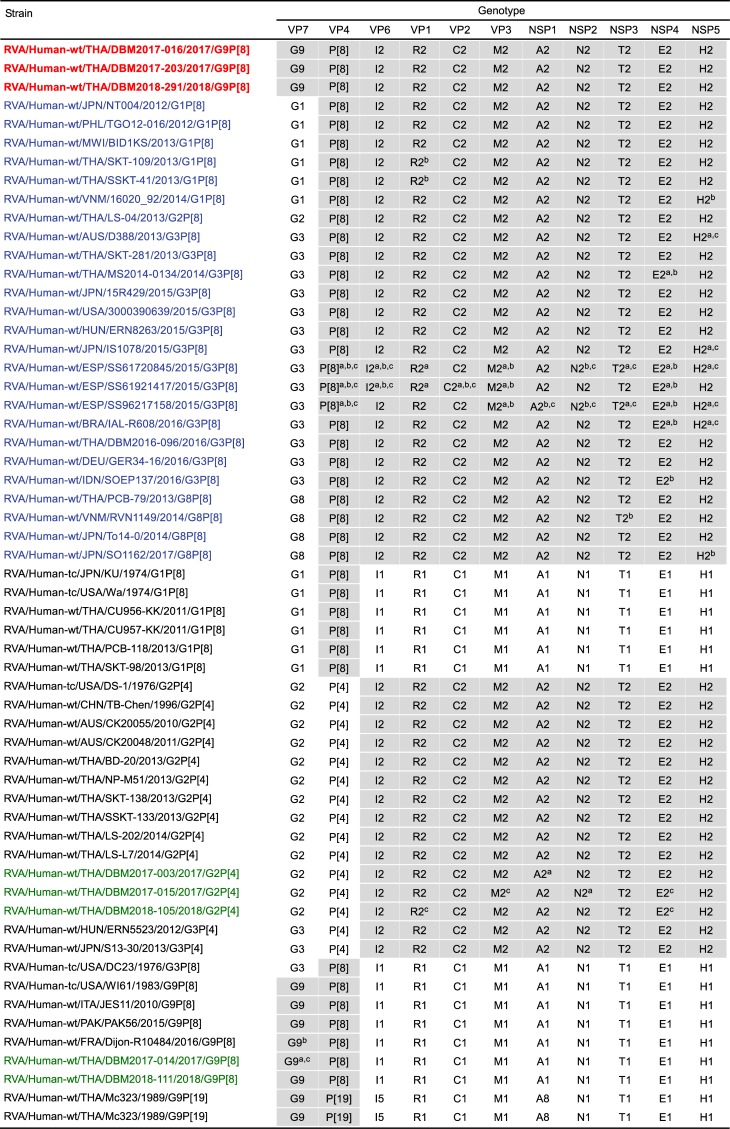
Genotype natures of the 11 gene segments of three Thai DS-1-like G9P[8] strains, DBM2017-016, DBM2017-203, and DBM2018-291, compared with those of selected HuRVA strains with known genomic constellations. The three Thai DS-1-like G9P[8] strains (DBM2017-016, DBM2017-203, and DBM2018-291) are shown in red, while the DS-1-like G1P[8] strains and their derivatives that have been reported are shown in blue. Three co-circulating DS-1-like G2P[4] strains (DBM2017-003, DBM2017-015, and DBM2018-105) and two Wa-like G9P[8] strains (DBM2017-014 and DBM2018-111) are shown in green. Gray shading indicates the gene segments with genotypes identical to those of the three Thai DS-1-like G9P[8] strains. ^a^The gene segments that are most similar to those of strain DBM2017-016. ^b^The gene segments that are most similar to those of strain DBM2017-203. ^c^The gene segments that are most similar to those of strain DBM2018-291.

The 11 genes of the three study strains, DBM2017-016, DBM2017-203, and DBM2018-291, were all assigned as G9-P[8]-I2-R2-C2-M2-A2-N2-T2-E2-H2 (Fig 1). The three strains were confirmed to have the G9P[8] genotype and a DS-1-like genetic backbone, as suggested on RT-PCR-based G/P genotyping and RNA electropherotyping, respectively (Tacharoenmuang et al., in preparation). Strains DBM2017-016, DBM2017-203, and DBM2018-291 were named RVA/Human-wt/THA/DBM2017-016/2017/G9P[8], RVA/Human-wt/THA/DBM2017-203/2017/G9P[8], and RVA/Human-wt/THA/DBM2018-291/2018/G9P[8], respectively, according to the guidelines for the uniformity of RVAs proposed by the RCWG. Comparison of the complete genotype constellations of the three study strains with those of other G9 and non-G9 HuRVA strains is shown in Fig 1. Except for the G genotype, the three strains had a unique genotype constellation (P[8]-I2-R2-C2-M2-A2-N2-T2-E2-H2), which is commonly found in emerging DS-1-like intergenogroup reassortant strains having the G1/3/8P[8] genotype [6, 10, 12–34]. On the other hand, only three of the 11 genes (VP4, VP6, and VP2) of these study strains appeared to be virtually identical (>99.4% identity), suggesting genomic variety among these three strains. The 11 gene segments of five locally circulating HuRVA strains, DBM2017-003, DBM2017-015, DBM2018-105, DBM2017-014, and DBM2018-111, were assigned as G2-P[4]-I2-R2-C2-M2-A2-N2-T2-E2-H2 (strains DBM2017-003, DBM2017-015, and DBM2018-105) and G9-P[8]-I1-R1-C1-M1-A1-N1-T1-E1-H1 (strains DBM2017-014 and DBM2018-111) (Fig 1), and thus they were named RVA/Human-wt/THA/DBM2017-003/2017/G2P[4], RVA/Human-wt/THA/DBM2017-015/2017/G2P[4], RVA/Human-wt/THA/DBM2018-105/2018/G2P[4], RVA/Human-wt/THA/DBM2017-014/2017/G9P[8], and RVA/Human-wt/THA/DBM2018-111/2018/G9P[8], respectively.

### Phylogenetic analyses

**Fig 2:**
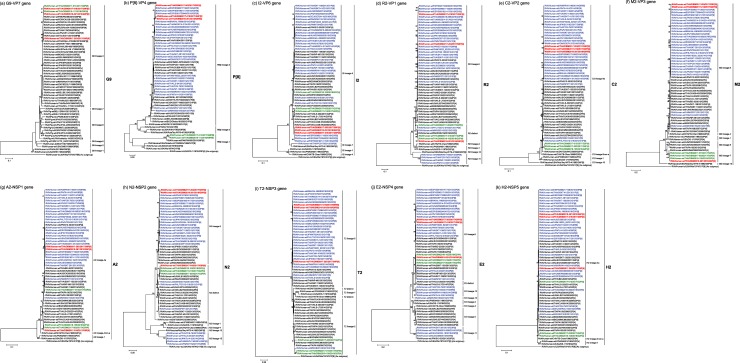
**Phylogenetic trees constructed from the nucleotide sequences of the G9-VP7 (a), P[**8**]-VP4 (b), I2-VP6 (c), R2-VP1 (d), C2-VP2 (e), M2-VP3 (f), A2-NSP1 (g), N2-NSP2 (h), T2-NSP3 (i), E2-NSP4 (j), and H2-NSP5 (k) genes of strains DBM2017-016, DBM2017-203, and DBM2018-291, and representative RVA strains.** In all the trees, the positions of the three Thai DS-1-like G9P[8] strains are shown in red, while those of other DS-1-like intergenogroup reassortant strains are shown in blue. Co-circulating strains, DBM2017-003, DBM2017-015, DBM2018-105, DBM2017-014, and DBM2018-111, are shown in green. Bootstrap values of <75% are not shown. Scale bars: 0.02 (b), 0.05 (h and i), 0.1 (a, c, d, e, g, and k), and 0.2 (f and j) substitutions per nucleotide.

The three study strains, DBM2017-016, DBM2017-203, and DBM2018-291, were further characterized by constructing phylogenetic trees using the full-length sequences for each of the 11 genes because phylogenetic analysis of RVA nucleotide sequences provides precise information on the origin of a given strain, and for tracing its evolutionary pattern, even within the same genotype [5, 41] (Fig 2A–2K). The nucleotide sequence identities between the three study strains and a representative close strain as to each gene are shown in Table 1.

**Table 1:** Nucleotide sequence identities (%) between three Thai DS-1-like G9P[8] strains, DBM2017-016, DBM2017-203, and DBM2018-291, and a representative closest strain as to each segment.

Gene	Study strain (nucleotide sequence identity)		
	RVA/Human-wt/THA/DBM2017-016/2017/G9P[8]	RVA/Human-wt/THA/DBM2017-203/2017/G9P[8]	RVA/Human-wt/THA/DBM2018-291/2018/G9P[8]
VP7	RVA/Human-wt/THA/DBM2017-014/2017/G9P[8]* (100%)	RVA/Human-wt/FRA/Dijon-R10484/2016/G9P[8] (99.8%)	RVA/Human-wt/THA/DBM2017-014/2017/G9P[8]* (99.9%)
VP4	RVA/Human-wt/ESP/SS61720845/2015/G3P[8] (99.5%)	RVA/Human-wt/ESP/SS61720845/2015/G3P[8] (100%)	RVA/Human-wt/ESP/SS61720845/2015/G3P[8] (99.6%)
VP6	RVA/Human-wt/ESP/SS61720845/2015/G3P[8] (99.6%)	RVA/Human-wt/ESP/SS61720845/2015/G3P[8] (99.7%)	RVA/Human-wt/ESP/SS61720845/2015/G3P[8] (99.8%)
VP1	RVA/Human-wt/ESP/SS61720845/2015/G3P[8] (99.6%)	RVA/Human-wt/THA/PCB-180/2013/G1P[8] (99.6%)	RVA/Human-wt/THA/DBM2018-105/2018/G2P[4]* (99.5%)
VP2	RVA/Human-wt/ESP/SS61921417/2015/G3P[8] (99.7%)	RVA/Human-wt/ESP/SS61921417/2015/G3P[8] (99.8%)	RVA/Human-wt/ESP/SS61921417/2015/G3P[8] (99.7%)
VP3	RVA/Human-wt/ESP/SS61720845/2015/G3P[8] (99.6%)	RVA/Human-wt/ESP/SS61720845/2015/G3P[8] (99.7%)	RVA/Human-wt/THA/DBM2017-003/2017/G2P[4]* (100%)
NSP1	RVA/Human-wt/THA/DBM2017-003/2017/G2P[4]* (99.9%)	RVA/Human-wt/ESP/SS96217158/2015/G3P[8] (99.6%)	RVA/Human-wt/ESP/SS96217158/2015/G3P[8] (99.7%)
NSP2	RVA/Human-wt/THA/DBM2017-015/2017/G2P[4]* (100%)	RVA/Human-wt/ESP/SS61720845/2015/G3P[8] (99.7%)	RVA/Human-wt/ESP/SS61720845/2015/G3P[8] (99.7%)
NSP3	RVA/Human-wt/ESP/SS61720845/2015/G3P[8] (99.7%)	RVA/Human-wt/VNM/RVN1149/2014/G8P[8] (99.6%)	RVA/Human-wt/ESP/SS61720845/2015/G3P[8] (99.8%)
NSP4	RVA/Human-wt/THA/MS2014-0134/2014/G3P[8] (99.3%)	RVA/Human-wt/THA/MS2014-0134/2014/G3P[8] (99.3%)	RVA/Human-wt/THA/DBM2017-015/2017/G2P[4]* (100%)
NSP5	RVA/Human-wt/ESP/SS61720845/2015/G3P[8] (99.8%)	RVA/Human-wt/JPN/NT004/2012/G1P[8] (99.7%)	RVA/Human-wt/ESP/SS61720845/2015/G3P[8] (99.8%)

Asterisks indicate locally circulating strains DBM2017-014 (G9P[8]), DBM2017-003 (G2P[4]), and DBM2017-015 (G2P[4]).

The VP7 genes of strains DBM2017-016 and DBM2018-291 showed the maximum nucleotide sequence identities (100 and 99.9%, respectively) with that of locally circulating human strain DBM2017-014 (G9P[8]) (Table 1 and Fig 1), and comparable identities (99.6 and 99.5%, respectively) with locally circulating human strain DBM2018-111 (G9P[8]). On phylogenetic analysis, strains DBM2017-016 and DBM2018-291 formed a cluster with these co-circulating Wa-like G9P[8] human strains within human-like G9 lineage-3, in which the majority of globally circulating G9 HuRVA strains cluster (Fig 2A). On the other hand, the VP7 gene of strain DBM2017-203 exhibited the highest nucleotide sequence similarity (99.8%) with contemporary French Wa-like G9P[8] human strain Dijon-R10484 [42] (Table 1 and Fig 1), and somewhat lower identities (99.4 and 99.1%, respectively) with Italy Wa-like G9P[8] human strain JES11 [43] and Japanese Wa-like G9P[8] strain UR14-16 [23]. Phylogenetically, strain DBM2017-203 was very closely related with strain Dijon-R10484 in a common branch with strains UR14-16 and JES11 within human-like G9 lineage-3, slightly away from the cluster comprising strains DBM2017-016 and DBM2018-291 (Fig 2A).

All of the VP4 genes of strains DBM2017-016, DBM2017-203, and DBM2018-291 showed the highest nucleotide sequence similarities (99.5–100%) with the cognate genes of Spanish equine-like G3P[8] strains (SS61720845, SS61921417, and SS96217158) [10] (Table 1 and Fig 1). On phylogenetic analysis, strains DBM2017-016, DBM2017-203, and DBM2018-291 were found to form a cluster with these Spanish equine-like G3P[8] strains in P[8] lineage-3 (Fig 2B).

All of the VP6 genes of strains DBM2017-016, DBM2017-203, and DBM2018-291 exhibited the highest nucleotide sequence identities (99.6–99.8%) with the VP6 genes of Spanish equine-like G3P[8] strains SS61720845 and SS61921417 (Table 1 and Fig 1), and comparable identities (99.6–99.8%) with Spanish equine-like G3P[8] strain SS96217158. On phylogenetic analysis, strains DBM2017-016, DBM2017-203, and DBM2018-291 were shown to form a cluster with these Spanish equine-like G3P[8] strains (Fig 2C).

The VP1 gene of strain DBM2017-016 showed the maximum nucleotide sequence identity (99.6%) with the cognate genes of Spanish equine-like G3P[8] strains SS61720845, SS61921417, and SS96217158 (Table 1 and Fig 1). On phylogenetic analysis, strain DBM2017-016 formed a cluster with these Spanish equine-like G3P[8] strains (Fig 2D). In contrast, the VP1 gene of strain DBM2017-203 exhibited the maximum nucleotide sequence identity (99.6%) with Japanese bovine-like G8P[8] strain SO1162 [44], Thai DS-1-like G1P[8] strains PCB-180, SKT-109, and SSKT-41 [21], and Vietnamese DS-1-like G1P[8] strain 16020_72 [45] (Table 1 and Fig 1). Phylogenetically, strain DBM2017-203 was found to be very closely related with strain SO1162 in a common branch with these DS-1-like G1P[8] strains from Thailand and Vietnam (Fig 2D). Furthermore, the VP1 gene of strain DBM2018-291 showed the highest nucleotide sequence similarity (99.5%) with locally circulating human strain DBM2018-105 (G2P[4]) (Table 1 and Fig 1), and comparable identity (99.4%) with locally circulating human strain DBM2017-015 (G2P[4]). Phylogenetically, strain DBM2018-291 formed a cluster with these co-circulating DS-1-like G2P[4] human strains (Fig 2D).

All of the VP2 genes of strains DBM2017-016, DBM2017-203, and DBM2018-291 showed the highest nucleotide sequence similarities (99.7, 99.8, and 99.7%, respectively) with the VP2 gene of Spanish equine-like G3P[8] strain SS61921417 (Table 1 and Fig 1), and comparable identities (99.6–99.7%) with Spanish equine-like G3P[8] strains SS61720845 and SS96217158. On phylogenetic analysis, strains DBM2017-016, DBM2017-203, and DBM2018-291 were shown to be very closely related with these Spanish equine-like G3P[8] strains (Fig 2E).

The VP3 genes of strains DBM2017-016 and DBM2017-203 showed the maximum nucleotide sequence identities (99.6 and 99.7%, respectively) with those of Spanish equine-like G3P[8] strains SS61720845, SS96217158, and SS96217158 (Table 1 and Fig 1). On phylogenetic analysis, strains DBM2017-016 and DBM2017-203 formed a cluster with these Spanish equine-like G3P[8] strains (Fig 2F). Conversely, the VP3 gene of strain DBM2018-291 exhibited complete nucleotide sequence identity (100%) with locally circulating human strains DBM2017-003 (G2P[4]) and DBM2017-015 (G2P[4]) (Table 1 and Fig 1), and comparable identity (99.9%) with locally circulating human strain DBM2018-105 (G2P[4]). Phylogenetically, strain DBM2018-291 was found to be very closely related these co-circulating DS-1-like G2P[4] human strains (Fig 2F).

The NSP1 gene of strain DBM2017-016 exhibited the highest nucleotide sequence identity (99.9%) with the cognate gene of locally circulating human strain DBM2017-003 (G2P[4]) (Table 1 and Fig 1), and somewhat lower identity (99.5%) with locally circulating human strain DBM2018-105 (G2P[4]). On phylogenetic analysis, strain DBM2017-016 formed a cluster with these co-circulating DS-1-like G2P[4] human strains (Fig 2G). On the other hand, the NSP1 genes of strains DBM2017-203 and DBM2018-291 showed the maximum nucleotide sequence similarities (99.6 and 99.7%, respectively) with Spanish equine-like G3P[8] strain SS96217158 (Table 1 and Fig 1), and comparable identities (99.5–99.6%) with Spanish equine-like G3P[8] strains SS61420845 and SS61921717. Phylogenetically, strains DBM2017-203 and DBM2018-291 were found to form a cluster with these Spanish equine-like G3P[8] strains in a common branch with several equine-like G3P[8] strains from different parts of the world (Fig 2G).

The NSP2 gene of strain DBM2017-016 exhibited complete nucleotide sequence identity (100%) with that of locally circulating human strain DBM2017-015 (G2P[4]) (Table 1 and Fig 1), and comparable identities (99.6 and 99.9%, respectively) with locally circulating human strains DBM2017-003 (G2P[4]) and DBM2018-105 (G2P[4]). On phylogenetic analysis, strain DBM2017-016 formed a cluster with these co-circulating DS-1-like G2P[4] human strains (Fig 2H). On the other hand, the NSP2 genes of strains DBM2017-203 and DBM2018-291 showed the highest nucleotide sequence identity (99.7%) with Spanish equine-like G3P[8] strains SS61720845 and SS96217158 (Table 1 and Fig 1), and comparable identity (99.6%) with Japanese equine-like G3P[8] strain IS1078 [22] and Spanish equine-like G3P[8] strain SS61921417. Phylogenetically, strains DBM2017-203 and DBM2018-291 were found to form a cluster with strain IS1078 in a common branch with these Spanish equine-like G3P[8] strains (Fig 2H).

The NSP3 genes of strains DBM2017-016 and DBM2018-291 showed the highest nucleotide sequence identities (99.7–99.8%) with the NSP3 genes of Spanish equine-like G3P[8] strains SS61720845 and SS96217156 (Table 1 and Fig 1), and comparable identities (99.6 and 99.7%, respectively) with Spanish equine-like G3P[8] strain SS61921417. On phylogenetic analysis, strains DBM2017-016 and DBM2018-291 formed a cluster with these Spanish equine-like G3P[8] strains (Fig 2I). In contrast, the NSP3 gene of strain DBM2017-203 exhibited the maximum nucleotide sequence identity (99.6%) with Vietnamese bovine-like G8P[8] strain RVN1149 [17] (Table 1 and Fig 1), and comparable identity (99.5%) with Japanese bovine-like G8P[8] strain SO1162. On phylogenetic analysis, strain DBM2017-203 was found to form a cluster with these Asian bovine-like G8P[8] strains (Fig 2I).

The NSP4 genes of strains DBM2017-016 and DBM2017-203 showed the maximum nucleotide sequence similarity (99.3%) with the cognate genes of equine-like G3P[8] strains from Thailand (MS2014-134) [46], Brazil (IAL-R608) [47], Indonesia (SOEP137) [32], and Spain (SS61720845, SS61921417, and SS96217158) (Table 1 and Fig 1), and comparable identities (98.7–99.2%) with equine-like G3P[8] strains from Hungary (ERN8263) [13], Japan (15R429 [20] and IS1078 [6]), Spain (SS98244047) [10], and the United States (3000390639) [28]. On phylogenetic analysis, strains DBM2017-016 and DBM2017-203 were found to be clustered near these equine-like G3P[8] strains from different parts of the world (Fig 2J). In contrast, the NSP4 gene of strain DBM2018-291 exhibited complete nucleotide sequence identity (100%) with locally circulating human strains DBM2017-015 (G2P[4]) and DBM2018-105 (G2P[4]) (Table 1 and Fig 1), and comparable identity (99.9%) with locally circulating human strain DBM2017-003 (G2P[4]). Phylogenetically, strain DBM2018-291 was shown to form a cluster with these co-circulating DS-1-like G2P[4] human strains (Fig 2J).

The NSP5 genes of strains DBM2017-016 and DBM2018-291 exhibited the highest nucleotide sequence identity (99.8%) with those of equine-like G3P[8] strains from Spain (SS61720845 and SS96217158), Australia (D388 [12]), Brazil (IAL-R608), and Japan (IS1078) (Table 1 and Fig 1), and comparable identity (99.6%) with equine-like G3P[8] strains from Thailand (MS2014-0134), Indonesia (SOEP137), Japan (15R429), and Spain (SS61921417). On phylogenetic analysis, strains DBM2017-016 and DBM2018-291 were found to be clustered near these and several DS-1-like intergenogroup reassortant strains from different parts of the world (Fig 2K). On the other hand, the NSP5 gene of strain DBM2017-203 showed the maximum nucleotide sequence similarity (99.7%) with Japanese DS-1-like G1P[8] strain NT004 [15], Japanese bovine-like G8P[8] strain SO1162, and Vietnamese DS-1-like G1P[8] strain 16020_92 [47] (Table 1 and Fig 1), and comparable identities (99.4–99.5%) with Thai DS-1-like G1P[8] strains SKT-109 and SSKT-41, Philippine DS-1-like G1P[8] strain TGO12-016 [33], and American equine-like G3P[8] strain 300390639. Phylogenetically, strain DBM2017-203 was closely related with these and several DS-1-like intergenogroup reassortant strains from different parts of the world (Fig 2K).

## Discussion

In this study, we determined and characterized the whole genomes of three unusual DS-1-like G9P[8] strains that have emerged in Thailand (strains DBM2017-016, DBM2017-203, and DBM2018-291). All the three DS-1-like G9P[8] strains exhibited a unique genotype constellation comprising a mixture of genogroup 1 and 2 genes: G9-P[8]-I2-R2-C2-M2-A2-N2-T2-E2-H2. On phylogenetic analysis, eight of the 11 genes of strain DBM2017-016 (VP4, VP6, VP1-VP3, and NSP3-NSP5) were found to be closely related to those of DS-1-like intergenogroup reassortant strains, while the remaining three (VP7, NSP1, and NSP2) were found to be closely related to locally circulating Wa-like G9P[8] and/or DS-1-like G2P[4] strains. Similarly, seven of the 11 genes of strain DBM2018-291 (VP4, VP6, VP2, NSP1-NSP3, and NSP5) were closely related to those of DS-1-like intergenogroup reassortant strains, while the remaining four (VP7, VP1, VP3, and NSP4) were closely related to locally circulating Wa-like G9P[8] and/or DS-1-like G2P[4] strains. Moreover, 10 of the 11 genes of strain DBM2017-203 (VP4, VP6, VP1-VP3, and NSP1-NSP5) were closely related to those of DS-1-like intergenogroup reassortant strains, while the remaining VP7 gene was closely related to contemporary Wa-like G9P[8] strains. Therefore, the three strains were suggested to be multiple reassortant viruses that had acquired the G9-VP7 genes from co-circulating human strains. Of note is that three (VP7, NSP1, and NSP2) and four (VP7, VP1, VP3, and NSP4) genes of strains DBM2017-016 and DBM2018-291, respectively, were assumed to have originated from locally circulating Wa-like G9P[8] and/or DS-1-like G2P[4] strains. Thus, the genomic diversity among Thai DS-1-like G9P[8] intergenogroup reassortant strains detected in the present study appeared to have been generated through additional reassortment with locally circulating RVAs [6, 10, 13, 16, 19, 22, 31, 44]. It should also be noted that the VP7 gene of strain DBM2017-203 was closely related to that of contemporary French Wa-like G9P[8] strain Dijon-R10484, however, epidemiological links to Thailand were missing. More genome data for global HuRVA strains are required for precise understanding of the evolution of DS-1-like intergenogroup reassortant strains in Asia and Europe. In any case, our data suggest a co-circulating pool of different DS-1-like G9P[8] strains in Thailand. Furthermore, a globally co-circulating pool of different equine-like G3P[8] strains has been suggested [26, 30], highlighting the remarkable potential of these emergent DS-1-like intergenogroup reassortant strains to evolve rapidly, probably through reassortment with locally circulating RVA strains.

The decreasing detection of common HuRVA strains such as Wa-like G1P[8] viruses and increasing detection of uncommon HuRVA strains such as DS-1-like intergenogroup reassortant viruses are an emerging concern in relation to HuRVA vaccine strategies. Although it is apparent that the current live-attenuated HuRVA vaccines (Rotarix (GlaxoSmithKline) and RotaTeq (Merck)) are highly effective against severe HuRVA disease, these vaccines might have put selective pressure on circulating HuRVA strains [6, 12, 46, 47]. HuRVA vaccines have not been introduced to the national immunization program in Thailand yet, but a monovalent Rotarix vaccine (G1P[8]) was introduced in Sukhothai province as a pilot study in 2011, in which the vaccine effectiveness for hospitalized RVA diarrhea was 88% (95%CI 76–94) [48]. Thus, it would be important to consider monitoring the evolution and circulation of emergent DS-1-like intergenogroup reassortant strains in the context of vaccination after the introduction of vaccines to the national immunization program. It would be important to perform RT-PCR-based genotyping for non-G/P gene(s) or PAGE in addition to genotyping for G/P genes for detection of unusual intergenogroup reassortant HuRVAs including the studied DS-1-like G9P[8] strains.

## Supporting information

S1 Table Sequence data for the 11 gene segments of eight Thai RVA strains, DBM2017-016, DBM2017-203, DBM2018-291, DBM2017-003, DBM2017-015, DBM2018-105, DBM2017-014, and DBM2018-111. (DOCX)
