# Supplementary material for: Full genome characterization of novel DS-1-like G9P[8] rotavirus strains that have emerged in Thailand
Source: PLoS One. 2020 Apr 22;15(4):e0231099. doi: 10.1371/journal.pone.0231099 (PMC7176146; doi:10.1371/journal.pone.0231099)
Supplement: S1 Table — (DOCX) [file pone.0231099.s001.docx]

**S1 Table. Sequence data for the 11 gene segments of eight Thai RVA strains, DBM2017-016, DBM2017-203, DBM2018-291, DBM2017-003, DBM2017-015, DBM2018-105, DBM2017-014, and DBM2018-111.**

| Study strain | Total reads^a^ |  | Gene | | | | | | | | | | |
| --- | --- | --- | --- | --- | --- | --- | --- | --- | --- | --- | --- | --- | --- |
|  |  |  | VP7 | VP4 | VP6 | VP1 | VP2 | VP3 | NSP1 | NSP2 | NSP3 | NSP4 | NSP5 |
| RVA/Human-wt/THA/DBM2017-016/2017/G9P[8] | 635,738 | Nucleotides; bp  (% coverage of the full-length) | 1056  (99.4%) | 2350  (99.6%) | 1355  (99.9%) | 3302  (100%) | 2684  (100%) | 2585  (99.8%) | 1563  (99.8%) | 1047  (98.9%) | 1066  (100%) | 740  (98.5%) | 802  (98.3%) |
|  |  | Deduced amino acids; aa  (% coverage of the full-length) | 326  (100%) | 775  (100%) | 397  (100%) | 1088  (100%) | 879  (100%) | 835  (100%) | 486  (100%) | 317  (100%) | 313  (100%) | 175  (100%) | 200  (100%) |
|  |  | Reads mapped to gene segment | 41,063 | 84,492 | 20,536 | 134,932 | 88,434 | 91,297 | 46,128 | 40,597 | 41,775 | 9,491 | 5,792 |
|  |  | Maximum depth of reads | 6,675 | 5,238 | 5,635 | 5,503 | 5,701 | 4,433 | 5,656 | 5,831 | 5,210 | 3,539 | 1,544 |
| RVA/Human-wt/THA/DBM2017-203/2017/G9P[8] | 620,353 | Nucleotide; bp  (% coverage of the full-length) | 1047  (98.6%) | 2351  (99.7%) | 1347  (99.3%) | 3300  (99.9%) | 2674  (99.6%) | 2585  (99.8%) | 1564  (99.9%) | 1047  (98.9%) | 1065  (99.9%) | 719  (95.7%) | 796  (97.5%) |
|  |  | Deduced amino acids; aa  (% coverage of the full-length) | 326  (100%) | 775  (100%) | 397  (100%) | 1088  (100%) | 879  (100%) | 835  (100%) | 486  (100%) | 317  (100%) | 313  (100%) | 175  (100%) | 200  (100%) |
|  |  | Reads mapped to gene segment | 16,391 | 71,839 | 19,644 | 132,184 | 79,610 | 95,797 | 47,576 | 42,459 | 44,198 | 8,185 | 4,485 |
|  |  | Maximum depth of reads | 3,802 | 5,674 | 5,958 | 5,997 | 5,093 | 4,561 | 6,086 | 6,290 | 5,781 | 3,421 | 1,608 |
| RVA/Human-wt/THA/DBM2018-291/2018/G9P[8] | 742,001 | Nucleotide; bp  (% coverage of the full-length) | 1045  (98.4%) | 2344  (99.4%) | 1314  (96.9%) | 3274  (99.2%) | 2617  (97.5%) | 2561  (98.8%) | 1486  (94.9%) | 1026  (96.9%) | 1047  (98.2%) | 724  (96.4%) | 816  (100%) |
|  |  | Deduced amino acids; aa  (% coverage of the full-length) | 326  (100%) | 775  (100%) | 397  (100%) | 1088  (100%) | 862  (98.1%) | 835  (100%) | 486  (100%) | 317  (100%) | 313  (100%) | 175  (100%) | 200  (100%) |
|  |  | Reads mapped to gene segment | 49,260 | 89,534 | 48,472 | 126,095 | 105,686 | 77,117 | 30,286 | 40,107 | 33,100 | 19,706 | 20,375 |
|  |  | Maximum depth of reads | 8,071 | 7,071 | 7,363 | 5,493 | 6,433 | 4,384 | 4,009 | 6,988 | 4,449 | 6,173 | 5,571 |
| RVA/Human-wt/THA/DBM2017-003/2017/G2P[4] | 846,427 | Nucleotide; bp  (% coverage of the full-length) | 1062  (100%) | 2359  (100%) | 1356  (100%) | 3294  (99.8%) | 2673  (99.6%) | 2590  (99.9%) | 1565  (99.9%) | 1047  (98.9%) | 1065  (99.9%) | 739  (98.4%) | 816  (100%) |
|  |  | Deduced amino acids; aa  (% coverage of the full-length) | 326  (100%) | 775  (100%) | 397  (100%) | 1088  (100%) | 879  (100%) | 835  (100%) | 486  (100%) | 317  (100%) | 313  (100%) | 175  (100%) | 200  (100%) |
|  |  | Reads mapped to gene segment | 40,701 | 91,082 | 22,641 | 174,489 | 115,915 | 98,001 | 49,190 | 35,794 | 49,603 | 7,420 | 3,523 |
|  |  | Maximum depth of reads | 5,542 | 7,850 | 6,506 | 7,484 | 7,316 | 5,355 | 7,295 | 7,049 | 6,274 | 3,502 | 1,022 |
| RVA/Human-wt/THA/DBM2017-015/2017/G2P[4] | 573,344 | Nucleotide; bp  (% coverage of the full-length) | 1061  (99.9%) | 2351  (99.7%) | 1347  (99.3%) | 3302  (100%) | 2673  (99.6%) | 2586  (99.8%) | 1566  (100%) | 1047  (98.9%) | 1065  (99.9%) | 727  (96.8%) | 797  (97.7%) |
|  |  | Deduced amino acids; aa  (% coverage of the full-length) | 326  (100%) | 775  (100%) | 397  (100%) | 1088  (100%) | 879  (100%) | 835  (100%) | 486  (100%) | 317  (100%) | 313  (100%) | 175  (100%) | 200  (100%) |
|  |  | Reads mapped to gene segment | 29,745 | 55,358 | 16,843 | 106,491 | 74,516 | 68,673 | 29,785 | 31,049 | 32,178 | 7,229 | 3,656 |
|  |  | Maximum depth of reads | 4,015 | 4,264 | 3,775 | 4,328 | 4,371 | 3,427 | 4,531 | 4,235 | 4,432 | 2,395 | 1,037 |
| RVA/Human-wt/THA/DBM2018-105/2018/G2P[4] | 661,764 | Nucleotide; bp  (% coverage of the full-length) | 1062  (100%) | 2349  (99.6%) | 1356  (100%) | 3291  (99.7%) | 2681  (99.9%) | 2582  (99.7%) | 1566  (100%) | 1047  (98.9%) | 1064  (99.8%) | 743  (98.9%) | 803  (98.4%) |
|  |  | Deduced amino acids; aa  (% coverage of the full-length) | 326  (100%) | 775  (100%) | 397  (100%) | 1088  (100%) | 879  (100%) | 835  (100%) | 486  (100%) | 317  (100%) | 313  (100%) | 175  (100%) | 200  (100%) |
|  |  | Reads mapped to gene segment | 31,177 | 59,824 | 22,750 | 97,969 | 74,812 | 70,321 | 35,268 | 30,151 | 31,929 | 11,820 | 7,774 |
|  |  | Maximum depth of reads | 3,629 | 3,755 | 3,398 | 4,487 | 4,450 | 3,422 | 4,214 | 3,825 | 3,772 | 3,127 | 1,589 |
| RVA/Human-wt/THA/DBM2017-014/2017/G9P[8] | 614,548 | Nucleotide; bp  (% coverage of the full-length) | 1059  (99.7%) | 2350  (99.6%) | 1348  (99.4%) | 3300  (99.9%) | 2734  (100%) | 2569  (99.2%) | 1553  (99.2%) | 1048  (99.0%) | 1071  (99.7%) | 741  (98.7%) | 649  (97.7%) |
|  |  | Deduced amino acids; aa  (% coverage of the full-length) | 326  (100%) | 775  (100%) | 397  (100%) | 1088  (100%) | 896  (100%) | 835  (100%) | 486  (100%) | 317  (100%) | 310  (100%) | 175  (100%) | 197  (100%) |
|  |  | Reads mapped to gene segment | 37,003 | 71,833 | 13,557 | 125,919 | 100,067 | 86,735 | 45,802 | 37,145 | 54,745 | 14,712 | 2,982 |
|  |  | Maximum depth of reads | 5,537 | 5,742 | 5,061 | 5,774 | 5,607 | 5,294 | 5,404 | 5,260 | 7,564 | 5,375 | 1,292 |
| RVA/Human-wt/THA/DBM2018-111/2018/G9P[8] | 799,888 | Nucleotide; bp  (% coverage of the full-length) | 1061  (99.9%) | 2350  (99.6%) | 1356  (100%) | 3292  (99.7%) | 2733  (99.9%) | 2541  (98.1%) | 1555  (99.9%) | 1023  (96.6%) | 1074  (100%) | 746  (99.3%) | 644  (97.0%) |
|  |  | Deduced amino acids; aa  (% coverage of the full-length) | 326  (100%) | 775  (100%) | 397  (100%) | 1088  (100%) | 896  (100%) | 835  (100%) | 486  (100%) | 317  (100%) | 310  (100%) | 175  (100%) | 197  (100%) |
|  |  | Reads mapped to gene segment | 57,042 | 82,601 | 17,880 | 150,639 | 116,851 | 89,952 | 50,827 | 44,848 | 63,412 | 18,455 | 4,400 |
|  |  | Maximum depth of reads | 9,485 | 5,907 | 6,236 | 6,700 | 6,889 | 6,067 | 6,061 | 6,190 | 8,679 | 6,168 | 1,838 |

^a^Sequence reads remaining after adapter trimming.
